# Lamotrigine-Induced Toxidermia

**DOI:** 10.1192/j.eurpsy.2026.11653

**Published:** 2026-07-31

**Authors:** K. Taleb, F. Laajili, B. Hana, Z. Bencharfa, F. El Omari

**Affiliations:** 1 Psychiatry, Arrazi hospital; 2 Razi Hospital; 3 Arrazi hospital, Salé, Morocco

## Abstract

**Introduction:**

Lamotrigine, widely used in bipolar and depressive disorders, may rarely cause severe cutaneous adverse reactions such as DRESS syndrome. Early recognition is crucial to prevent severe complications.

**Objectives:**

To report a case of lamotrigine-induced DRESS syndrome and emphasize the importance of early detection and careful monitoring to prevent severe complications.

**Methods:**

**Case Report:** A 19-year-old female treated for recurrent depressive disorder was started on lamotrigine (50 mg/day). Six weeks later, she developed a diffuse pruritic maculopapular rash, fever (38.5°C), cervical lymphadenopathy, and fatigue.

Laboratory tests showed eosinophilia (1200/μL) and moderate hepatic cytolysis (AST 85 U/L, ALT 92 U/L).

The RegiSCAR score suggested probable DRESS. Lamotrigine was promptly discontinued, and systemic corticosteroids were introduced, leading to rapid improvement and resolution of skin lesions within 10 days.

**Results:**

**Discussion** DRESS is a rare but potentially fatal drug-induced reaction, typically occurring 2–8 weeks after exposure. The risk increases with rapid dose escalation or valproate co-administration.

Management includes immediate drug withdrawal, symptomatic treatment, and systemic corticosteroids in severe cases.

**Conclusions:**

Ce cas souligne la nécessité d’une surveillance étroite lors de l’instauration du traitement par lamotrigine.

Tout signe d’alerte cutané ou systémique doit inciter à une évaluation urgente afin de prévenir les complications graves liées au syndrome DRESS.

**Disclosure of Interest:**

None Declared

